# Migraine associated with auditory-vestibular dysfunction

**DOI:** 10.1016/S1808-8694(15)30611-X

**Published:** 2015-10-18

**Authors:** Renato Cal, Fayez Bahmad

**Affiliations:** 1 Otorhinolaryngologist, researcher of the Otology Department, Massachusetts Eye & Ear Infirmary.; 2 Otorhinolaryngologist, graduation doctoral student, UnB Medical School, assistant physician of the Otorhinolaryngology and Head & Neck Surgery Department, Brasilia University Hospital, researcher of the Otology Department, Massachusetts Eye & Ear Infirmary.

**Keywords:** migraine, vestibulocochlear system, vertigo

## Abstract

The association between hearing and balance disorders with migraine is known since the times of the ancient Greeks, when Aretaeus from Cappadocia in 131 B.C, made an accurate and detailed description of this occurrence during a migraine episode. We present a broad review of migraine neurotological manifestations, using the most recent publications associated with epidemiology, clinical presentation, pathophysiology, diagnostic methods and treatment for this syndrome.

**Aim:**

to describe the clinical entity: “Migraine associated with auditory-vestibular dysfunction” in order to help otorhinolaryngologists and neurologists in the diagnosis and management of such disorder.

**Final Remarks:**

There is a strong association between neurotological symptoms and migraine, and the auditory-vestibular dysfunction-associated migraine is the most common cause of spontaneous episodic vertigo (non-positional). Symptoms may vary broadly among patients, making it a diagnostic challenge to the otorhinolaryngologist. This entity usually presents with positional or spontaneous vertigo spells, lasting for seconds or days, associated with migraine symptoms. A better understanding of the relationship between central vestibular mechanisms and migraine mechanisms, besides the discovery of ionic channel disorders in some cases of migraine, ataxia and vertigo, may lead to a better understanding of migraine pathophysiology associated with audio-vestibular disorder.

## INTRODUCTION

The association between hearing/balance disorders and migraine has been recognized since ancient Greece, when in 131 a.C., Aretaeus of Cappadocia described precisely and in detail the occurrence of both conditions during a migraine crisis.^1^ In 1861, Prosper Ménière published a classical paper describing the symptoms of Ménière Syndrome (MS) in migraine patients.^2^ In 1873, Liveing reported a clear association between vertigo and migraine, which has since been also reported by various authors: Gowers et al., 1907; Symonds et al., 1926; Graham et al., 1968; and Kayan et al., 1984. These papers defined the concept that vertigo, hearing loss and tinnitus were part of the symptoms that some migraine patients presented.^3^, ^4^, ^5^, ^6^

In 1961, Bickerstaff, introduced the Basilar Migraine concept, which is characterized by occipital headache with signs or symptoms of cranial nerve and/or brainstem dysfunction, such as: disorders of vision, vertigo, ataxia, speech disorders, tinnitus, and sensory alterations on extremities.^7^

Various types of balance disorders have been reported during headache crises in migraine patients, such as rotational vertigo, positional vertigo, dizziness, intolerance to head movement, and other less common findings.^4^, ^8^, ^9^, ^10^, ^11^, ^12^, ^13^, ^14^, ^15^, ^16^

Classical migraine patients presenting kinetosis in childhood or as adults appear to be more susceptible to migraine crises.^4^, ^16^, ^21^.

Reported auditory disorders include phonophobia (intolerance to loud noise) and hyperacusis (abnormal noise sensitivity), first described by Tissot in 1778 (cited by Sachs et al., 1970), which may be related to headache-induced stress.^1^

Currently, otologists and neurologists have received patients presenting a clinical picture of migraine-like headaches, episodes of dizziness, at times even vertigo, aural fullness, auditory symptoms and tinnitus. These patients have led to a description of a new clinical entity, which has been given various names, including: migraine-associated dizziness, migraine-related dizziness, migraine-related vertigo, migrainous vertigo, migraine-anxiety related dizziness and migraine-associated cochleovestibular dysfunction.^6^, ^14^, ^22^, ^23^, ^24^

Given the paucity of references on this theme in Portuguese, he authors have chosen the name “migraine-related auditory-vestibular dysfunction” The reason for this is that many patients present vestibular findings that differ from the true concept of vertigo; symptoms may range from a feeling of unbalance to instability, vertigo, and in some cases even cochlear disorders such as tinnitus, intermittent dysacusis and auricular fullness.

**Figure 1:**
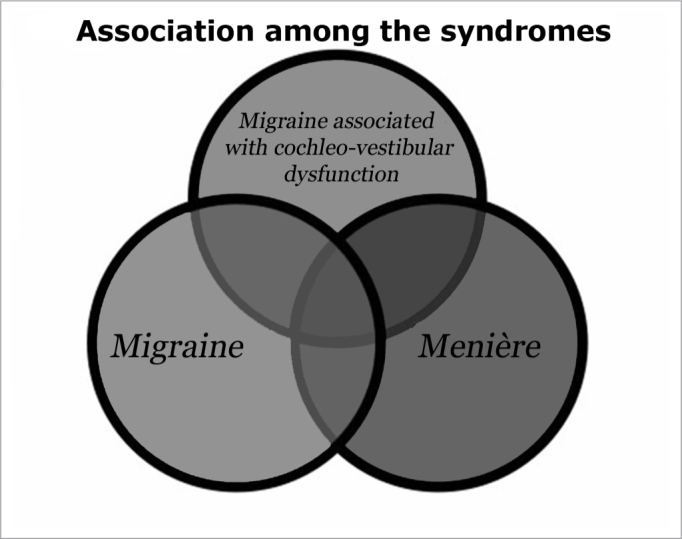
Relations among the syndromes.

The pathophysiology of migraine-related auditory-vestibular symptoms has not been fully clarified, but its clinical manifestation is varied.^23^ These patients usually present symptoms ranging from episodes of dizziness to acute vertigo and constant unbalance, sensorineural hearing loss, tinnitus, aural fullness and intermittent dysacusis. These symptoms are often mistaken with the classical symptoms of MS. It is, therefore, a syndrome that lies between migraine with aura and MS; the differential diagnosis between these three entities is often a major challenge (Figure 1 and Table 1), requiring significant healthcare professional experience and knowledge about their clinical, diagnostic and therapeutic aspects.^22^, ^25^, ^26^, ^27^

## OBJECTIVES

The purpose of this study was to describe the clinical entity “migraine-related auditory-vestibular dysfunction” to support otorhinolaryngologists and neurologists in the diagnosis and clinical management of this syndrome.

The authors reviewed the literature and the clinical experience of a North-American tertiary reference hospital for otoneurological disorders, looking at signs and symptoms and vestibular clinical findings for the diagnosis and treatment of these patients.

### Epidemiology

Epidemiological data have underlined the increasing importance of migraine-related auditory-vestibular dysfunction in recent years. Recent papers have shown that migraine is one of the diseases with the highest morbidity worldwide; it affects about 4-6.5% of men and 11.2-18.2% of women both in the USA and in Europe.^26^, ^28^ A correlation between migraine-related auditory-vestibular dysfunction and MS shows that the incidence of MS in the USA is about 5-15 for each 100,000 individuals (0.015%). Based on these numbers, we may assume that if 1% of migraine-related auditory-vestibular dysfunction patients presented sufficient episodes of vertigo to seek medical help, physicians would diagnose 15 times more patients with this entity compared to MS.^25^ The international medical literature pays insufficient attention to migraine-related auditory-vestibular dysfunction, suggesting that this condition is frequently not diagnosed. Lipton et al. (2002) studied a sample of 4,376 patients and revealed that migraine affects individuals in their most economically productive age group, namely 30-39 years (25.7%), 40-49 years (24.4%) and 18-29 years (22.3%), which underlies the importance of correctly diagnosing and treating this condition.^28^

This connection is even more evident in epidemiological studies of migraine patients: 28 to 36% of patients present associated dizziness, and 25 to 26% present vertigo.^26^ Similarly, 36% of patients that complain of dizziness may fit into the clinical criteria for migraine, and 61% of patients with vertigo of unknown causes may also fit into the clinical criteria for migraine.^24^ In a case-control study of 10 patients, Furman et al. revealed that dizziness and vertigo occur in 54% of migraine patients and in only 30% of patients with headaches of other origins, such as tension-type headaches,^23^, ^29^ reinforcing the hypothesis of comorbidity between migraine and cochleovestibular disorders.

**Chart 1:**
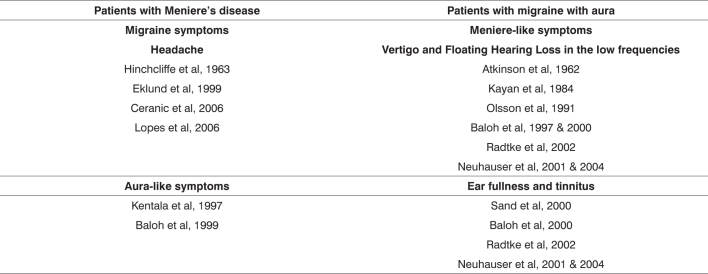
Association between symptoms and syndromes.

### Clinical and Diagnostic Findings

Many individuals, and even healthcare professionals, still think that migraine is unilateral, pulsatile headache associated with visual and auditory disorders, and usually associated with nausea and vomiting. This concept remains correct and is the best know aspect of migraine with aura, according to the International Headache Society (IHS) criteria that were described in 1988.^30^ Currently, however, medical researchers have widened the scope of migraine, describing it as a global sensorial perceptive alteration in an individual. Any sensorial perception-related symptom may be described, including hearing, olfactive, visual, tactile, gustatory and postural disorders.^6^, ^23^, ^28^

### Differentiating migraine with aura from migraine with auditory-vestibular disorders

Duration of headache is the first point to note when differentiating migraine-related auditory-vestibular dysfunction from classical migraine with aura. In classical migraine, symptoms may last from a few seconds to 60 minutes, while in migraine-related auditory-vestibular dysfunction cases, symptoms generally last hours, days or even months. Adult patients with migraine-associated vestibular dysfunction usually report spontaneous or positional vertigo, occasionally initiating as spontaneous vertigo and eventually becoming positional vertigo.6 An important point is that the duration of this type of positional vertigo differs from that seen in benign paroxysmal positional vertigo (BPPV). Patients with migraine-related cochleovestibular dysfunction usually describe vertigo while the head is in the due trigger position, contrary to what occurs in BPPV, in which vertigo lasts only a few seconds. Intolerance to movement is another characteristic feature of migraine-related auditory-vestibular dysfunction, which is very similar to the clinical findings of kinetosis (feeling of unbalance, illusion of movement and nausea worsened by head movements).^31^ Visual vertigo induced by movie screens, by lighting such as that found in stores and shopping malls and by computer screens is typical of migraine-related auditory-vestibular dysfunction.

Vertigo attacks may vary widely, lasting from minutes to two hours (46.4%), and many hours or weeks (30.4%).^22^ Some individuals may require weeks to months to recover fully from an attack of vertigo. Such attacks may occur at intervals of days, weeks, months, or even years. Differentiating migraine-related auditory-vestibular dysfunction from migraine with aura may be more difficult when clinical findings last between 5 and 60 minutes,.

A careful clinical history is the best weapon for physicians to diagnose migraine-related auditory-vestibular dysfunction. Neuhauser et al published the clinical criteria for diagnosing this entity in a classical paper based on the IHS guidelines for diagnosing migraine.^32^ This paper, which has probably contributed most to the diagnostic standardization of that clinical entity, defined criteria for migraine-related vestibular disease for the defined and probable forms. These authors used the method of assessing three groups of patients: the first group included outpatient subjects presenting with vestibular disorders (n=200), the second group included patients with migraine (n=200) as defined by IHS criteria, and the third group included patients from the orthopedics unit of the same hospital that belonged to the same age group as group 1 patients (control group / n=200). After analyzing these groups, the authors proposed the criteria for classifying migraine-related auditory-vestibular dysfunction that are presented on Table 2.

**Chart 2:**
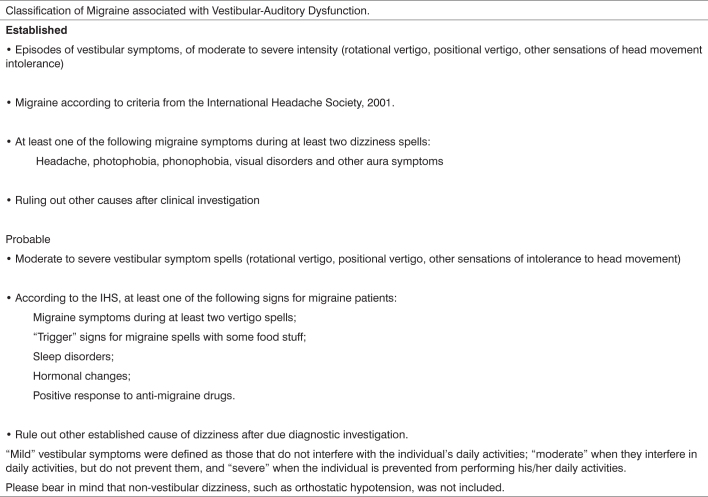
Classification of Migraine associated with Vestibular-Auditory Dysfunction.

These criteria have been adopted by most of the authors and international clinical units in this area in an attempt to standardize the diagnosis of migraine-related auditory-vestibular dysfunction.^14^, ^15^, ^16^, ^21^, ^22^, ^23^, ^26^, ^33^, ^35^ More recently, Marcus et al. developed a questionnaire based on Neuhauser's criteria to serve as a screening tool for patients with vestibular conditions in which a diagnosis of migraine-related vestibular disease is considered.^33^

Many authors have applied vestibular and auditory testing in patients diagnosed with migraine-related auditory-vestibular dysfunction in an attempt to establish a pattern for vestibular findings that would support the diagnosis of this condition. Another aim of vestibular testing would be to differentiate migraine-related auditory-vestibular dysfunction from the initial stages of MS.^16^, ^20^, ^22^, ^23^, ^36^, ^37^, ^38^

### Differentiating migraine-related auditory-vestibular dysfunction from Ménière's syndrome (MS)

Battista analyzed the audiometric findings of 76 patients with migraine-related auditory-vestibular dysfunction n and 34 patients diagnosed with MS. Results revealed that although there were descriptions in the literature of auditory disorders in patients with migraine-related auditory-vestibular dysfunction, most of them had normal hearing, different from patients with MS (including intermittent hearing loss as part of the typical clinical picture of MS).^22^

Furman et al. studied patients with migraine-related auditory-vestibular dysfunction by using a battery of vestibular tests and found altered vestibular-spinal function tests in posturography. Balance was increased in patients with migraine-related auditory-vestibular dysfunction compared to both control groups, particularly in the Sensory Organization Test 4, 5 and 6 (Equitest®); the same patients had normal oculomotor movements and caloric responses during vertigo-free periods,^23^, ^29^ and more frequently altered rotatory chair test results compared to abnormal findings on electronystagmography (ENG).^20^, ^37^

Dimitri et al. analyzed multiple variables in an attempt to establish a difference between migraine-related auditory-vestibular dysfunction and MS based on vestibular testing. The authors concluded that decreased vestibular responses in caloric testing and altered rotatory chair testing were significant in 91% of cases when differentiating migraine-related auditory-vestibular dysfunction and MS.38 In summary, there appears to be no typical pattern in vestibular testing for establishing a diagnosis of migraine-related auditory-vestibular dysfunction, which reinforces even further the need for a carefully taken clinical history. Vestibular testing such as ENG, the rotatory chair test, electrocochleography (ECoG), and vestibular myogenic evoked potentials (VEMP) may support a diagnostic hypothesis raised in the clinical history. Progressive hearing loss is still the beset method for differentiating migraine-related auditory-vestibular dysfunction and MS.

### Treatment

Having made a diagnosis of migraine-related auditory-vestibular dysfunction, the next step is counseling of patients. In general, patients with this condition respond positively to well-established therapy for migraine.^21^, ^39^, ^40^ The first step in controlling the symptoms is to persuade patients to adopt the so-called “migraine lifestyle,” which includes dietary changes, regular exercise, and regular sleep. Dietary changes include decreasing or eliminating the use of aspartame, chocolate, caffeine and alcohol.^40^ It is also essential to exercise regularly, to decrease stress and to improve sleep.^39^ If these behavioral measures are unsuccessful, medication may be used for controlling vertigo episodes. There are the migraine-suppressing drugs (benzodiazepines, beta-blockers, tricyclic antidepressants, etc.) and the migraine-aborting drugs (mainly sumatriptan). Migraine-aborting drugs are an excellent option for treating migraine; however, in migraine-related auditory-vestibular symptoms, such drugs are generally not used because crises of vertigo usually occur as a headache aura.^13^ Thus, migraine-suppressing drugs are more used in otoneurology.

Possibly the most relevant data in the therapy of migraine-related auditory-vestibular symptoms were published in 2002 in a retrospective study that revealed that over 72% of patients improved or had complete remission of symptoms after step-by-step treatment was introduced.^21^ This treatment consists initially of changing the diet, followed by migraine-suppressing drugs such as low-dose tricyclic antidepressants, calcium channel blockers and beta-blockers.

The efficacy of drugs used in migraine-related auditory-vestibular dysfunction therapy is directly related with the ability of such drugs in combating or aborting headaches.^13^ Clinical randomized, prospective, double-blind, placebo-controlled studies for comparing the efficacy of various drugs in the treatment exclusively of migraine-related auditory-vestibular symptoms have not yet been published in the literature. Thus, otoneurologists use their personal experience in managing this syndrome.

The authors emphasize the idea that, when managing correctly headaches associated with auditory-vestibular symptoms, physicians should be aware of side-effects and reactions to drugs that otorhinolaryngologists do not prescribe regularly in everyday practice. It is therefore wise to begin with the lowest pharmacological dose.

The first step in treatment is to start with behavioral measures such as an adequate diet, good sleeping habits, regular physical exercise, and a decrease in stress levels during at least one month. Drug therapy is initiated if patients continue with regular episodes of migraine-related auditory-vestibular symptoms. Preferred drugs are tricyclic antidepressants, especially nortryptiline (10 mg/day) at night before sleeping, to minimize the main side effects (drowsiness and xerostomia). The initial dose should be maintained for two weeks, after which it is increased if migraine crises persist. Most of the patients benefit from a dose between 30 and 70 mg/day without presenting side effects.

Beta-blockers are the second drugs of choice for the authors, especially propanolol. The initial dose is 40 mg/day; most patients end up taking around 80mg/day. It is important to note that most of the patients with a diagnosis of migraine-related auditory-vestibular dysfunction are young women; these patients tend to present arterial hypotension, which makes beta-blockers an added risk. It is clear that therapy for each patient should be approached individually, taking into account factors such as age, sex, comorbidities, etc., so that the lowest dose capable of controlling the disease may be used, thus decreasing significant side effects.

Reploeg et al. demonstrated that 100% of migraine-related auditory-vestibular dysfunction patients reported some improvement in vertigo and unbalance after initiating one of these therapies.^21^ Another study involving 53 migraine-related auditory-vestibular dysfunction patients revealed that the efficacy of drugs in controlling vestibular symptoms was directly proportional to their headache-relieving ability.^13^ A current frequently debated theme in the treatment of this condition is the true importance of vestibular rehabilitation therapy. Various authors have published papers demonstrating the benefits of vestibular rehabilitation therapy in migraine-related auditory-vestibular dysfunction patients.^34^, ^39^, ^41^, ^42^.

## FINAL COMMENTS

Otoneurologists worldwide have studied migraine-related auditory-vestibular dysfunction, given its clinical manifestations that are similar to those found in a variety of other otoneurological diseases, particularly MS.

Being a recently described syndrome, most otorhinolaryngologists are not prepared for diagnosing this condition, which should be part of the differential diagnosis of vertigo and of the management of migraine patients.
